# MicroSweat: A Wearable Microfluidic Patch for Noninvasive and Reliable Sweat Collection Enables Human Stress Monitoring

**DOI:** 10.1002/advs.202204171

**Published:** 2022-12-03

**Authors:** Shaghayegh Shajari, Razieh Salahandish, Azam Zare, Mohsen Hassani, Shirin Moossavi, Emily Munro, Ruba Rashid, David Rosenegger, Jaideep S. Bains, Amir Sanati Nezhad

**Affiliations:** ^1^ BioMEMS and Bioinspired Microfluidic Laboratory Department of Biomedical Engineering University of Calgary Calgary Alberta T2N 1N4 Canada; ^2^ Stressynomics Hotchkiss Brain Institute Cumming School of Medicine University of Calgary Calgary Alberta T2N 1N4 Canada; ^3^ Department of Mechanical and Manufacturing Engineering University of Calgary Calgary Alberta T2N 1N4 Canada; ^4^ Department of Physiology and Pharmacology University of Calgary Calgary Alberta T2N 1N4 Canada; ^5^ International Microbiome Centre Cumming School of Medicine Health Sciences Centre University of Calgary Calgary Alberta T2N 1N4 Canada; ^6^ Department of Chemical and Petroleum Engineering University of Calgary Calgary Alberta T2N1 N4 Canada; ^7^ Department of Civil Engineering University of Calgary Calgary Alberta T2N1 N4 Canada; ^8^ SenseSi Co. Calgary Alberta T2L 1Y8 Canada

**Keywords:** biomarkers, cortisol, human stress, human sweat, microfluidics, MicroSweat, self‐powered capillary, wearable devices

## Abstract

Stress affects cognition, behavior, and physiology, leading to lasting physical and mental illness. The ability to detect and measure stress, however, is poor. Increased circulating cortisol during stress is mirrored by cortisol release from sweat glands, providing an opportunity to use it as an external biomarker for monitoring internal emotional state. Despite the attempts at using wearable sensors for monitoring sweat cortisol, there is a lack of reliable wearable sweat collection devices that preserve the concentration and integrity of sweat biomolecules corresponding to stress levels. Here, a flexible, self‐powered, evaporation‐free, bubble‐free, surfactant‐free, and scalable capillary microfluidic device, MicroSweat, is fabricated to reliably collect human sweat from different body locations. Cortisol levels are detected corresponding to severe stress ranging from 25 to 125 ng mL^−1^ averaged across multiple body regions and 100–1000 ng mL^−1^ from the axilla. A positive nonlinear correlation exists between cortisol concentration and stress levels quantified using the perceived stress scale (PSS). Moreover, owing to the sweat variation in response to environmental effects and physiological differences, the longitudinal and personalized profile of sweat cortisol is acquired, for the first time, for various body locations. The obtained sweat cortisol data is crucial for analyzing human stress in personalized and clinical healthcare sectors.

## Graphical abstract

This paper presents a flexible, self‐powered, skin‐interfaced, surfactant‐free microfluidic patch (MicroSweat) for reliable sweat collection and Chrono sampling of sweat from different body locations and chemical analysis of sweat cortisol stress biomarker. MicroSweat facilitates the potential benefit of measuring stress levels using the cortisol concentrations of the armpit sweat. It also signifies personalizing sweat cortisol for longitudinal and daily effects.

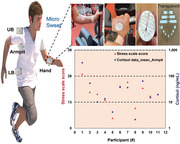

## 1 Introduction

Generating an appropriate physiological stress response is essential for survival and hemostasis. Inappropriate or prolonged stress responses, however, have negative consequences on the brain and body.^[^ ^1^ ^]^ These pathological stress responses have an enormous impact on the quality of life. They impact cognition, decision making, and social interactions and contribute to the progression of many diseases, such as neurodegenerative diseases and cancer.^[^ ^2^ ^]^ Understanding how stress affects physical and mental health requires a reliable approach to monitor this internal state from the outside of the body.^[^ ^3^ ^]^ Wearable smartwatches provide noninvasive monitoring of physiological parameters that may indicate changes in stress state. These parameters, however, are not direct stress signals; thus, changes do not directly reflect internal state changes.

The non‐invasive and accurate detection and quantification of human stress levels require direct measurement of stress biomarkers such as cortisol and troponin hormones, and neuropeptide Y (NPY), and epinephrine neurotransmitters.^[^ ^4^ ^]^ In humans, stress increases circulating levels of cortisol. This increase is also reflected as an increase in cortisol release from the sweat glands in the skin.^[^ ^5^ ^]^ Compared to other biofluids, sweat provides an advantage for continuous collection by wearable devices with minimal human intervention.^[^ ^6^ ^]^ The technologies for quantitative and longitudinal detection of stress biomarkers in sweat are in their infancy.

Considering the importance of cortisol monitoring, recent investigations on various electrochemical cortisol sensors^[^ ^7^ ^]^ show that significant challenges and technical hurdles have remained in the research and development of cortisol monitoring devices. Most of the current wearable sensors that contained electrodes on a flexible substrate were directly in contact with the skin to measure the cortisol response.^[^ ^8^ ^]^ Although they are simple structures that do not require a complicated fabrication process for creating sweat‐collection features on the device, the temporal resolution of their measured cortisol is often poor.^[^ ^9^ ^]^ This is due to environmental artifacts such as evaporations and contamination issues, and discontinuous sweat flow, which causes bubble reading signals, mixing of new and old sweat, and losing dynamic monitoring of sweat for temporal variation in its composition. In addition, since sweat is not sampled but sensed directly on the skin, the skin topography, skin temperature^[^ ^10^ ^]^ and pH^[^ ^11^ ^]^ may interfere considerably with the measurement results. Furthermore, due to the lack of a mechanism to actively remove sweat from the sensor, analytes both from sweat and the skin diffuse throughout the measured volume, leading to inaccurate results.

Therefore, a reliable sweat collection device should be supplemented for the applicability of these devices to accurately measure stress levels and shape future sweat cortisol monitoring devices. Most wearable sweat‐collecting microfluidic devices used silicone elastomers and lithography approaches for chip fabrication.^[^ ^12^ ^]^ However, the hydrophilic property of these materials is primarily temporal.^[^ ^13^ ^]^ Also, these materials often have a high affinity to many vitamins, hormones, and other potential biomarkers, adversely affecting their reliability for intact sample collection and/or biomarker sensing.^[^ ^12c^ ^]^ In addition, the material preparations and lithography processes require multistep processing, which is time‐consuming and costly. Other researchers have embedded two layers of elastomeric microfluidic channels, including the internal hydrophilic layer made of polyurethane (PU) and the outer layer made of silicone elastomers such as Ecoflex,^[^ ^14^ ^]^ polydimethylsiloxane (PDMS), or a combination of both.^[^ ^15^ ^]^ This is a significant challenge that must be overcome to effectively fabricate a microfluidic device with stable hydrophilic properties without adding further material layers and chemical steps to the fabrication or complicating the design.

We designed and fabricated a disposable, thin, flexible, and self‐powered skin‐interfaced microfluidic device (MicroSweat) using a fast processable one‐step laser cutting technique. Numerical modeling based on resistive networks was developed to optimize the geometry and structure of the microchannels, functional valves, and storage fibers and to control bubble‐free sweat flow and storage. MicroSweat is mechanically robust with a bendable structure that can be easily mounted and safely sealed on the skin surface of different body locations. An autonomous capillary design helped sweat flow based on only gland secretory pressure. It ensured a controlled amount of collected sweat from multiple adjacent points on the skin during the physical activity on treadmills. Incorporating thin‐film materials with a smart combination of surfactant‐free hydrophilic and hydrophobic properties and specific fibers enabled i) rapid uptake of the sweat into the system, ii) leakage‐free collecting and storing the sweat, iii) the preserved concentration of biomolecules and chemically intact sweat biomarkers, iv) increasing the volume of collected sweat up to 120 µL, and v) sequential storage of the sweat generated at different time points during the human test (Chrono sampling). The extracted sweat samples from the storage fibers were subject to ex‐situ cortisol analysis. The analysis between cortisol concentration and the standard stress tests were performed to investigate the sweat cortisol ranges in groups of different sex and age on different parts and sides of the body while considering longitudinal and personalized effects. Notably, the wearable MicroSweat patch was fabricated in a process compatible with the scale production of microfluidic devices. MicroSweat can be used in medicine for clinical and personalized health monitoring and in sport science to improve the performance of athletes.

## 2 Results and Discussion

### 2.1 Thin, Soft Microfluidic Devices for Sweat Sampling

**Figure 1:**
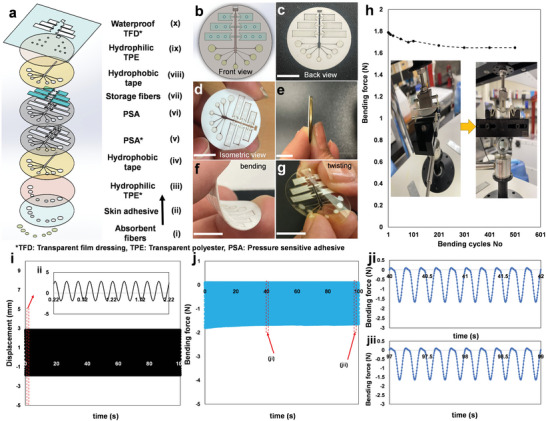
A schematic representation of the wearable MicroSweat microfluidic patch. a) The patch contains multiple thin layers of polymers, fibers, and adhesives. It collects sweat from the skin using absorbent fibers and guide it toward the storage chambers via hydrophilic coatings on transparent polyester (TPE). An exploded view of the assembled MicroSweat patch, including b) the top, c) the back, and d) the isometric view. e) The rational view for the patch thickness. f,g) Digital images of the flexibility of the assembled nontransplant and transparent MicroSweat during bending and twisting. Scale bars: 10 mm. Cyclic bending tests on the MicroSweat patch for 500 cycles of bending‐releasing with 5 Hz cycle frequency for a bending radius of 33 mm and a bending angle of 67°. h) The result of bending force over 500 cycles indicates the mechanical stability and flexibility of MicroSweat. i) Cyclic displacement graph with inset graph in (i‐i) showing a magnified view for a short time. j) Cyclic bending force measurement during bending cycling tests with inset figures in (j‐i) and (j‐ii), showing the magnified view in the mid‐ and final cycles, respectively.

The wearable MicroSweat patch was developed for reliable sweat collection and storage and ease of fabrication using laser cutting, assembly, and bonding of multilayers of flexible polymer sheets and fibers. **Figure** **1**a shows a schematic view of the different layers of the MicroSweat. Figure 1b–d shows the schematic view of the assembled MicroSweat in three different fronts, back, and isometric views. The thickness of MicroSweat is around 230 µm, while considering the skin adhesives (80 µm), the total thickness of the wearable is about 310 µm (Figure 1e). Figure 1f,g demonstrates flexibility in the bending and twisting ability of wearable MicroSweat when using non‐transparent and transparent PSA.

Figure 1h–j shows the stability of MicroSweat during 500 bending‐releasing cycles. The bending force reduces slightly at the first cycles because of the positioning of MicroSweat and the hysteresis effect owing to the viscoelasticity of the polymer. However, the hysteresis effect vanishes after a few cycles, given that the bending force magnitude change is negligible. The displacement of 5 mm applied during the bending‐releasing cycles is shown in Figure 1i,ii and the corresponding bending force measurement is presented in Figure 1j. Figure 1ji,jii indicates the stability of MicroSweat during the bending test at the mid‐and last cycles.

The laser cutter parameters were optimized for each layer of material to meet a) the smoothness needed for the surface of channels, b) the repeatability and reproducibility of the channels, and c) the minimal channel size obtainable using laser cutting. Table S1, Supporting Information, provides the optimal set of parameters for cutting each of the layers of MicroSweat. Figure S1a‐d, Supporting Information, shows that the variation between the channel widths in Ch1 to Ch7 is <±25 µm, indicating high reproducibility of creating microchannels of the wearable MicroSweat patches.

The wearable MicroSweat patch and its compartment's detailed configuration, including inlet reservoirs (RVs), delay and stop valves, channels (Ch), vents, storage chambers, and inlet and storage fibers, are shown in Figure S2, Supporting Information. It comprises a symmetrical geometry that absorbs the sweat from different locations on the skin using circular absorbent fibers (cellulose fibers). The sweat is drawn from the skin into MicroSweat through the inlet ports and cellulose fibers, followed by its capillary flow into the inlet channels. Prior to reservoir saturation (it has a small volume of 3–6 µL depending on the size of the reservoirs), sweat is drawn into the channels using the capillary force, making the inlet reservoirs act as self‐powered sweat pumps in the system and minimize the hydraulic pressure. The capillary forces control the sweat flow rate within the MicroSweat with no or minimal dependency on the sweat generation rate on the skin and its variations amongst people. The minimum achievable channels width was 125 ± 25 µm upon optimizing the laser parameters (see Figure S2b–g, Supporting Information).

**Figure 2:**
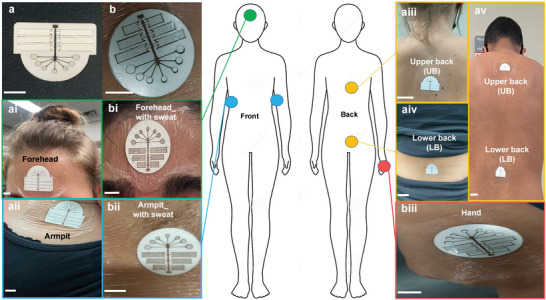
The wearability of MicroSweat patch on various body locations and its use for monitoring sweat dynamics and chemo stress signal analysis. a,b) Various configurations of the wearable MicroSweat patch mounted on different body locations before and after sweat collection from the forehead in (a‐i) and (b‐i), armpits in (a‐ii) and (b‐ii), hand in (b‐iii), and lower and upper backs in (a‐iii), (a‐iv), and (a‐v). The fibers look darker when they are soaked with sweat. Scale bars: (a), (b), (a‐i), (b‐i), (a‐ii), (b‐ii), (b‐iii): 10 mm; (a‐iii), (a‐iv), (a‐v): 30 mm.

**Figure 3:**
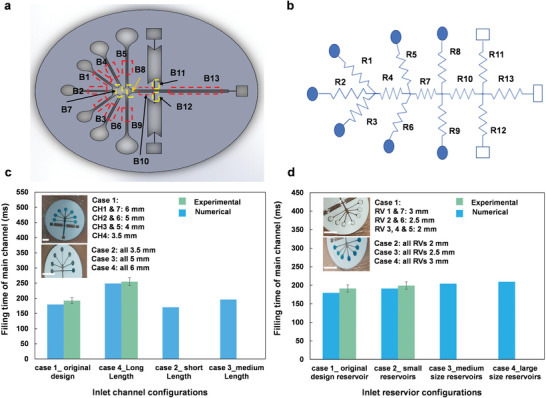
Electric circuit analogy model for MicroSweat and a comparison between experimental results and numerical modeling for the sweat flow in MicroSweat. a) Different branch of the patch, and b) a corresponding electrical equivalent circuit model of MicroSweat. The effect of c) different inlet channel and d) inlet reservoir configurations on the filling time of the main channel. For experiments in (c), (d), only two cases are considered to compare with the numerical results. Scale bars: 5 mm.

The MicroSweat patch is a single integrated unit containing various firmly assembled layers (Figure S3a,b, Supporting Information). The patch is attached to the skin by removing the carrier of the adhesive layer (as one of the MicroSweat layers) and the waterproof layer at the very bottom of the patch (Figure S3b,c, Supporting Information). Figure S3d, Supporting Information, shows a schematic side view of MicroSweat when it is attached to the skin by the adhesive and entirely sealed by the waterproof layer. Following the sweat generation on the skin, it flows into the inlet reservoirs and pushes the air toward the vents located at the upper surface of the patch. In addition, the insulating adhesive layer separates the skin from the microfluidic layers, eliminating or minimizing the effect of skin temperature on the collected sweat constituents. **Figure** **2**a,b shows two configurations of wearable MicroSweat patch and its attachment to different body locations, including hands, forehead, backs, and armpits. The sealing condition of MicroSweat mounted on hands and forearms during its bending and stretching is shown in Figure S4a–f and Movie S1, Supporting Information. The configuration of MicroSweat with the distributed branches used in the numerical model is shown in **Figure** **3**a. To implement an electric circuit analogy model, the microchannels in the capillary MicroSweat patch were converted to an equivalent resistance in an electrical circuit (Figure 3b). Each resistance represents a microchannel and is presented with a capital letter R. In each junction, the resistances are enumerated by the channels following a contour clockwise direction. Geometric information of the channels based on the resistance number in an equivalent electric circuit and the static contact angle measured for the top and bottom walls (hydrophilic transparent polyester (TPE)) and the left and right pressure sensitive adhesive (PSA) walls are presented in Table S2, Supporting Information.

To investigate the effect of microchannel geometries on the function of MicroSweat, the storage chambers were removed and replaced with two hydrophobic rectangular chambers with side vents. Given that every change in the microchannel dimensions alters both the flow resistance and capillary pressure (see Section S3 and Figure S5a,b, Supporting Information), one way to reduce the resistance without decreasing the capillary pressure is to use multiple parallel microchannels with different lengths.^[^ ^16^ ^]^ The length of the inlet channels and the size of the inlet reservoirs were accordingly changed to adjust the flow resistance while keeping the capillary pressure constant and the delay time for different channels to simultaneously collect the sweat by a plurality of inlet channels. The numerical model and experimental data show that the shortest filling time is achieved for cases 1 and 2 as opposed to cases 3 and 4 (Figure 3c,d). Although the filling time for case 2 in Figure 3c is shorter than in case 1, the plurality mechanism is dominant in case 1. This mechanism resulted in a faster filling of the storage chambers with increased sweat volume availability.

The effect of inlet channels width on the filling time of the main channel (Ch8) at the outlet is studied using experimental testing of the Microsweat patch (Figure S6, Supporting Information). Decreasing microchannel size from above 450 µm to <200 µm in width reduces the filling time for the main channel by 15 ms. Herein, two factors oppose each other, i) capillary forces and ii) flow resistance. Although the capillary driving force increases by a decrease in channel width, the flow resistance increases. The fluidic resistance increases monotonically during microchannel filling (replacing the air with the sweat).^[^ ^16^ ^]^ The more the volume of the microchannel network is filled with sweat, the slower would be the flow rate of sweat through the channel, assuming a constant rate of sweat generation on the skin.^[^ ^17^ ^]^ In smaller channel sizes, the capillary pressure is superior to the flow resistivity, which causes a shorter filling time.

**Figure 4:**
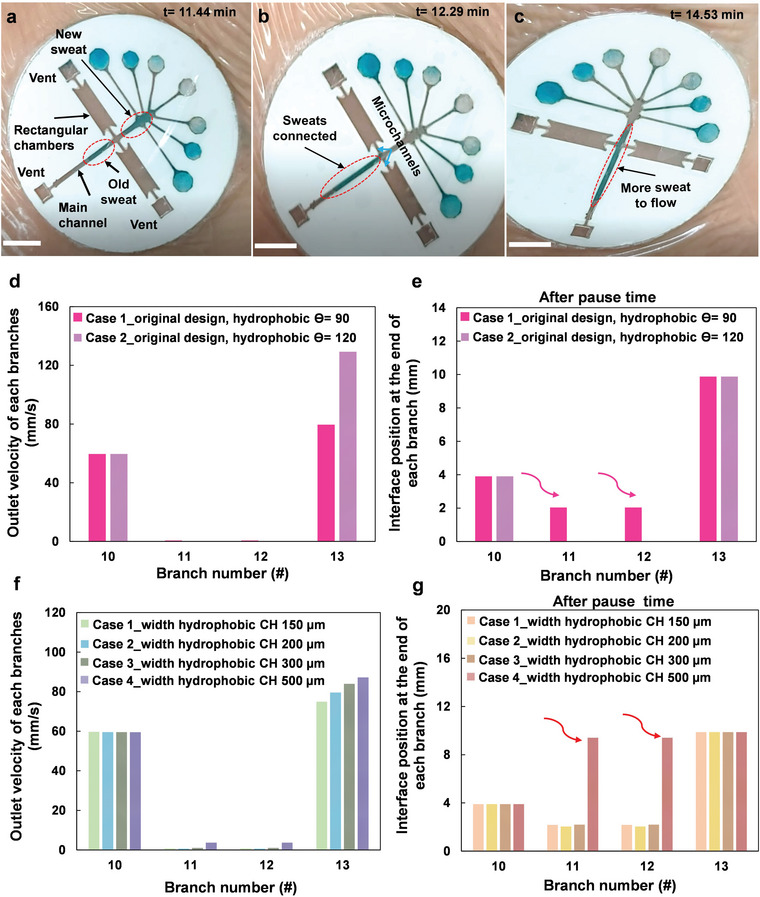
The illustration of stop valves performance during MicroSweat human tests with empty side rectangular chambers and bubble‐free sweat flow into the patch. a) Sweat passes the inlet microchannels and continues flowing downstream of the main channel while the bubble is removed from the side rectangular storage chambers via their vents. b,c) The freshly generated sweat connects to the previously generated sweat in the main channel without entering the storage chambers. The numerical modeling results for the hydrophobicity effect of side channels on the sweat velocity and interface position after pause time when comparing different contact angles in (d) and (e), and for the effect of microchannel width at the inlets of hydrophobic chambers in (f) and (g), respectively. Scale bars: 5 mm.

A delay is involved at the junction between the main channel and the inlet microchannels, which boosts the plurality effect where the inlet microchannels are filled separately but independently. The flow in the main channel is delayed until a few of the inlet microchannels contribute to appropriate sweat volume collection. The trigger (delay) valves embedded within each rectangular storage chamber's inlet, control the sweat flow into each storage chamber (**Figure** **4**a–c). Meantime, all bubbles flowing through the inlet channels into the main channel run out through the trigger valves and the storage chambers. This enables bubble‐free sweat flow downstream of the main channel where future biosensors would be embedded.

Figure 4a–c shows that there is an air bubble trapped between the old and new sweat, indicating the complete removal of the old sweat where there was a delay between old and new sweat generation for a participant. Two parts of the patch contribute to the removal of the old sweat from the skin: 1) the cellulose fibers embedded at the inlet reservoirs, which absorb all the sweat on the skin and conduct it to the reservoirs and channels, and 2) capillary force of the channel network which transports the sweat inward until all the old sweat in the reservoirs is drained toward the downstream channels and replaced with the new sweat. Therefore, storage fibers facilitate the collection of sweat generated at different time intervals. The older sweat is collected by the earlier storage fibers while the newer sweat is stored in the downstream storage fibers enabling the separation of the old and new sweat.

Given the reliability of the numerical models, tested, and verified for several microfluidic geometries and constitutions, numerical modeling was performed to study the effect of hydrophobic surfaces (two different contact angles 90^o^ and 120^o^) on the function of delay valves. The sweat velocity at the outlet is almost zero at branches 11 and 12 (side channels) for both contact angles 90^o^ and 120^o^ (Figure 4d). Only the valves with lower hydrophobicity are opened (Figure 4e), allowing the sweat to flow into the storage chambers. Figure 4f,g presents that sweat velocity at the outlet is almost zero for all microchannels widths tested, except for the largest microchannel width of 500 µm where the delay valves open. This function of delay valves was further used to engineer the sequential filling of storage chambers, wherein the size of chambers was adjusted to tune the flow resistance and, thus, the sequence of sweat flow into each chamber. Once the sweat passes the delay valve, it is absorbed by the embedded fibers within the storage chambers. The flow rate of the sweat is controlled by the wicking rate specified for each type of fiber. The delay valves and fibers' soaking capacity are configured to control the sweat flow rate and storage sequence. This exclusive approach can be further used for investigating the dynamics of target biomarkers released into the sweat during an hour of physical activity.

The MicroSweat patch also comprises several vents, one for the whole system downstream of the main channel and others to draw air bubbles out of the sweat in the storage chambers. The vents located on top of the storage chambers are <300 µm in diameter to prevent sweat evaporation (based on the Langmuir's free evaporation equation in vacuum^[^ ^11^, ^18^ ^]^ (see Section S4, Supporting Information) and help preserve the molecular contents of the sweat in the stored fibers. These components cooperate to remove air bubbles and provide a smooth laminar flow downstream of the main channel, thus providing continuous sweat flow into the patch. The removable fibers used for sweat storage are made of nitrocellulose fibers or glass microfibers with different wicking rates and volume capacity for sweat collection depending on the needs (see Figure S7, Supporting Information).

Furthermore, a saturation test was performed to verify the preservation of sweat content stored in the storage fibers at their saturation points. For this test, microfluidic patches were fabricated using transparent PSAs (see Section S5, Supporting Information). This fiber saturation confirms the preservation of sweat molecule concentrations in these fibers.

### 2.2 A Chrono Sampling of Sweat

**Figure 5:**
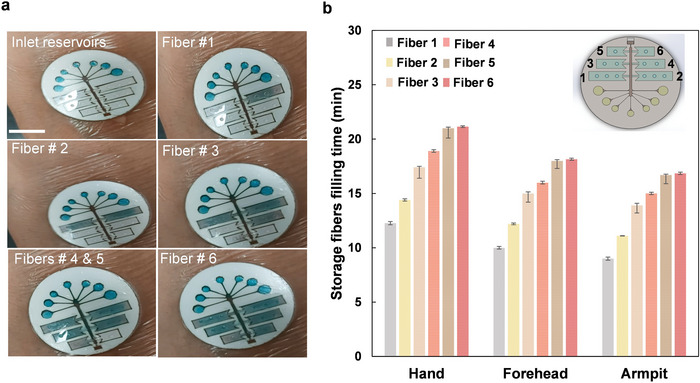
An illustration of Chrono sampling of the human sweat by wearable MicroSweat. a) Images of time‐dependent filling of the nitrocellulose fibers when collecting sweat from the hand. The error bar refers to three replication tests. Scale bar: 10 mm. b) The filling time of nitrocellulose storage fibers when collecting the sweat from the hand, forehead, and armpit. The error bar refers to three replication tests.

The digital images of the time‐dependent filling of the patch, mounted on hands, from the inlet reservoirs to the storage fibers #1 to #6 are shown in **Figure** **5**a. To real‐time trace the sweat flow into the storage chambers during the field testing, a blue dye was dried on the skin prior to mounting the patch on the skin. Figure 5b depicts the filling time of the nitrocellulose fibers in different storage chambers for the wearable MicroSweat patches mounted on the hand, forehead, and armpit. Regardless of the variation in the initiation of sweat generation at different body locations, the filling time intervals between the storage chambers show that the sweat is generated continuously from the body. The interval time of up to 2 min ± 20 s for larger fibers, 1.25 min ± 15 s for medium fibers, and up to 3 s for the smaller fibers indicates the reliable and repeatable pattern for Chrono sampling of the sweat. The order of filling the storage chambers with the sweat relies on the fluid resistance around the delay valves at the entrance of each storage chamber adjusted by the geometrical change, wherein the inlet (entrance) channels differ in size with a width difference of 100 ± 25 µm. The wider the entrance channel, the lower the channel resistance, and the faster the opening of delay valves to allow sweat flow into the storage chambers. This design configuration controls the order of filling the storage chambers from the bottom chambers to the top and in the side direction from the left chambers to the right. We further conducted a computational simulation to verify the sequential filling of the storage chambers with the results depicted in Figure S9 a,b, Supporting Information and described in Section S6, Supporting Information.

The Chrono sampling of the sweat in this work using flow resistance and capillary pressure differences is comparable to the few works in the literature referring to Chrono sampling of sweat,^[^ ^12^, ^13^, ^19^ ^]^ wherein the sweat was rapidly analyzed for a certain biomarker. We reduced the number of microfluidic elements, including valves, and simplified the design.

A video clip, Movie S2, Supporting Information, provides better insight into the sequential filling of the MicroSweat patch with sweat while it is mounted on the hands during in‐situ human sweat collection.

### 2.3 Intra‐Individual and Inter‐Individual Variations in Human Sweat Dynamics

**Figure 6:**
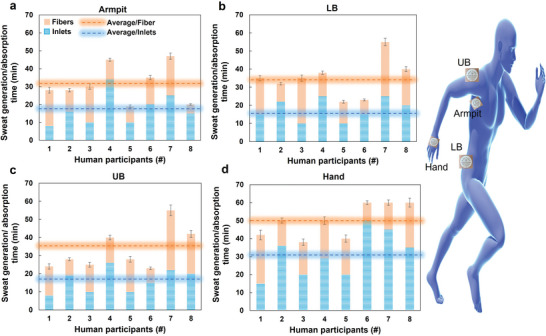
The dynamics of sweat generation tested on eight human participants when mounting the wearable MicroSweat patches simultaneously on different body locations. a) armpit, b) lower back (LB), c) upper back (UB), and d) hand. In the figure's insets, (a) the orange and blue dotted lines represent the average time values for sweat generation at the inlet reservoirs and the sweat storage at the storage fibers, respectively.

The MicroSweat patches with glass microfibers were mounted simultaneously on four different body locations (armpits, lower back (LB), upper back (UB), and hands). The participants were subject to a representative walking test on a treadmill. The initiation time of sweat generation (at the inlet reservoirs) and the time of storage (storage fibers) within MicroSweat were recorded using live video recording, and the results are shown in **Figure** **6**a–d. For the armpit sweat collection, the minimum time the sweat generates in all the inlets to the time all fibers collect sweat varies between 7 and 47 min with an average collection time of 31.5 min for different participants (Figure 6a). For the LB sweat collection, this time varies between 10 and 55 min, with an average time of 33.1 min (Figure 6b). Similarly, for the UB locations, this time is between 7 and 55 min with an average time of 35 min (Figure 6c), and for the hand sweat collection, it is within 15–60 min with an average time of 50 min (Figure 6d). The relatively long collection period is only applied to the tests aiming to collect high sweat volume (about 120 µL) for further biomarker discovery. In the future, biosensor‐integrated wearable patches would require a much smaller sweat volume (5–10 µL), significantly reducing the testing period to about 7–15 min.

The results of on‐human sweat collection tests show that there is an intra‐individual variation in sweat generation and collection as well as inter‐individual variation depending on the body location. When comparing the average time of sweat generation, the armpit body location has the highest rate of sweat generation, followed by LB and UB, with an almost two‐minute time difference. Having multiple inlet reservoirs, besides having the benefit of collecting sweat from different locations on the skin, can help with faster sweat collection due to the plurality effects of several inlet reservoirs.

### 2.4 Chemical Analysis of Sweat Cortisol in Human trials

Detection of cortisol as a stress biomarker in sweat is at the forefront of wearable stress detection devices currently in development.^[^ ^20^ ^]^ A thorough investigation is needed to quantify sweat cortisol in human subjects of different sex and age, collected from different body positions and at different time points, activity levels, and health status.

Further analysis is needed to unravel the correlation between cortisol concentration in the sweat and other biofluids. Such information would be crucial in the coming years to enable medical professionals to diagnose and monitor psychological disorders in a personalized manner.^[^ ^20^ ^]^ With more valid data available from sweat cortisol levels, specifically from eccrine and apocrine glands, the sweat biofluid can become an industry‐standard cortisol measurement method rather than only an alternative or auxiliary approach for quantifying stress hormone dynamics.^[^ ^6e^ ^]^

#### 2.4.1 Sweat Cortisol in Human trials: Effects of Sex, Age, Body locations, and Body sides

**Figure 7:**
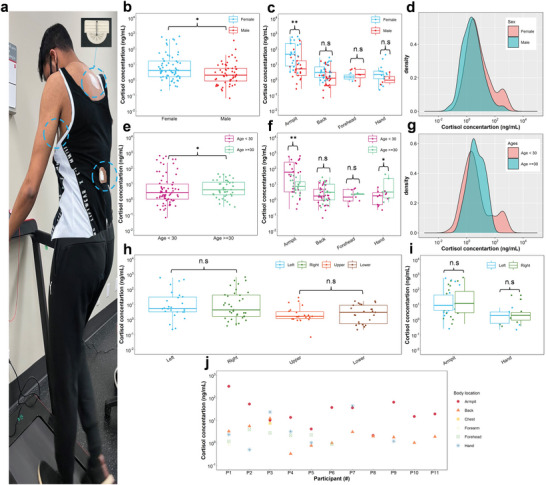
Sweat cortisol comparison between different group of individuals at different locations and sides of the body. a) human subject wearing MicroSweat at different body locations during treadmill walking. b) Statistical box plots of sweat cortisol concentration considering sex difference in different body locations, and c) in all body parts, and d) its density distribution plot. E) Statistical box plots for the effect of age difference on sweat cortisol concentration in all body parts, f) separately in different body locations, and g) its density distribution plot. h) Statistical box plot for sweat cortisol concentration collected from different body sides (left, right, upper, and lower) and i) separately for the left and right sides of the armpits and hands. *p < 0.05, and **p ≤  0.01, and n.s. not significant for two‐tailed unpaired *t*‐test. j) A map of sweat cortisol concentration between different human subjects and body locations (personalized sweat cortisol data).

Sweat biomarker information from both apocrine and eccrine glands^[^ ^21^ ^]^ facilitates the creation of accurate sweat‐based wearable diagnostic systems and could provide reliable sweat cortisol concentrations.^[^ ^6e^ ^]^ **Figure** **7**a shows that the wearable MicroSweat patch developed in this study can simultaneously collect human sweat from different body locations, including the sweat produced by eccrine and apocrine glands. This enables a fair comparison between different cortisol data of the human skin.

The t‐test analysis (Section S7, Supporting Information) of cortisol detected in the sweat collected from all body parts shows a significant difference in cortisol concentration between male and female subjects according to the mean value of sweat cortisol data (*p*‐value < 0.05 in 95% confidence interval) (Figure 7a). Except for the forehead, in both apocrine and eccrine sweat glands, sweat cortisol concentration is higher in females versus males, which may be attributed to the larger pituitary gland volume in females as the primary regulatory organ of cortisol.^[^ ^22^ ^]^ Moreover, adenylate cyclase, a second messenger released by the pituitary gland, promotes different levels of sweat secretion in women versus men.^[^ ^23^ ^]^

When comparing different body locations, the mean value difference in cortisol concentration is more highlighted in the armpit areas, showing a *p*‐value ≤ 0.01 among male and female groups (Figure 7b). This agrees with the findings by Tu. et al.^[^ ^24^ ^]^ and Pearlmutter et al.^[^ ^6e^ ^]^ who concluded that the average cortisol concentrations in the apocrine areas are larger than in the eccrine areas. However, they reported higher sweat cortisol concentrations in males than in females. Similar to the two studies above, the sweat cortisol difference between males and females is more highlighted for apocrine sweat than eccrine sweat.

Despite the mean value difference in cortisol concentration between males and females, the cortisol concentrations have similar distributions between males and females (Figure 7c). The detailed results of *t*‐tests and *F*‐tests (Section S7, Supporting Information) and their *p*‐values for cortisol concentration measured from all body parts and separately for different body locations are tabulated in Tables S3 and S4, Supporting Information.

Comparing the cortisol concentrations of two different age groups (< 30 years and ≥ 30 years), shown in Figure 7d, indicates a significant difference in their sweat cortisol levels (*p*‐value < 0.05; statistical analysis done using *F*‐test and *t*‐test; see Table S5, Supporting Information). The results show a slight increase in median cortisol concentrations with increasing age. However, a larger sweat cortisol distribution is observed for ages below 30 when comparing the density distribution plot of cortisol for the two age groups (Figure 7e). The difference in regional body locations highlights variations of cortisol concentrations between the two age groups in the armpit areas with a *p*‐value < 0.01 and hand areas with a *p*‐value < 0.05 (Figure 7f; see Table S6, Supporting Information, for more details on *t*‐test analysis).

There is no other study comparing sweat cortisol levels in different ages, but the cortisol concentration analysis in salivary biofluid in the literature shows that the increase in the salivary cortisol concentration is more prominent in aged women (>60) than young women (<30).^[^ ^25^ ^]^ Similarly, during adolescence and beyond the age of 50, a gradual increase in salivary cortisol was observed for both males and females, which was argued to be attributed to the onset of puberty.^[^ ^26^ ^]^ In contrast, studying the effect of age on salivatory cortisol concentration by Nicolson et al.^[^ ^27^ ^]^ showed no change in the hypothalamic‐pituitary‐adrenal (HPA) activity and the subsequent release of the steroid hormone cortisol from the outer cortex of the adrenal gland until the ages of over 70. In addition, they found no difference in salivary cortisol levels between males and females at all ages except for higher cortisol levels in old women than in old men. The diversity between male and female cortisol responses is evident from different studies, though there is no census for sweat and salivary cortisol levels. Specifically, reports on sweat cortisol levels are rare. Therefore, with higher supporting evidence in future experiments, further analysis of more sample numbers can provide more robust support for these claims.

While recent studies have been focused on evaluating sweat cortisol levels primarily for eccrine areas,^[^ ^9^ ^]^ an estimation of the symmetry among sides of the body for each body location simultaneously has not been investigated yet. In this study, cortisol concentration among individuals is assessed between different body sides at a similar region on each side, including the left and right sides of the armpits, hands, and upper and lower sides of the back. Figure 7g maps the sweat cortisol concentration difference between identical parts but opposite sides of the body. The effect of the body side on sweat cortisol concentration is insignificant for both left and right sides, including the armpits and hands (Figure 7h), and for the upper and lower parts of the body (back). This is indicated by a *p*‐value > 0.05, all with a 95% confidence level (see Tables S7 and S8, Supporting Information, for the *t*‐test and *F*‐test analysis). Although the number of sweat glands and sweat rate might differ at each side of the body, they seem to mostly affect the sweat ions (excluding ions like Na^+^)^[^ ^28^ ^]^ with a minimal effect on other biomarkers.

Regardless of the population‐level data discussed above, sweat biomarker data collected using wearable devices would benefit from obtaining personalized data. A map of sweat cortisol concentrations measured from the sweat collected for different body locations of different individuals is shown in Figure 7i. Comparing the data among individuals proposes that each individual may have a personalized signature of cortisol concentrations in various body parts.

#### 2.4.2 Sweat Cortisol in Human Trials: Personalized Longitudinal and Daily Effect

**Figure 8:**
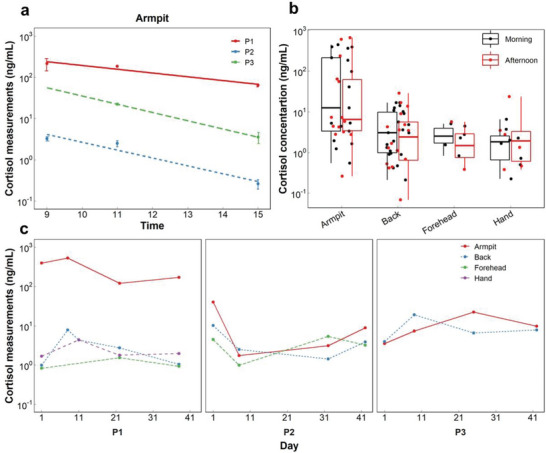
Personalized longitudinal and daily observation of sweat cortisol concentration of different body locations, including armpit, back, forehead, and hand. a) Statistical box plots of the sweat cortisol concentration and a comparison between morning and afternoon for different body locations and b) its daily variations for armpit locations of different healthy human participants (P1–P3) at 9 AM, 11 AM, and 3 PM. c Sweat cortisol change over a month for various body locations and three healthy human subjects (P1–P3) and all at the same time in the morning.

Cortisol level is known to undergo a diurnal cycle modulated by the circadian rhythm, with a higher amount in the morning, a lower amount in the afternoon, and the lowest amount at night.^[^ ^8^, ^29^ ^]^ The same temporal change of cortisol concentration was also reported for all biofluids, such as saliva, serum, and sweat.^[^ ^8^, ^30^ ^]^ The daily variation in cortisol concentration from 9 AM to 3 PM and at three different times of day is observed individually for three human subjects measured in armpit areas (**Figure** **8**a). Most body locations also demonstrate this variation, with a mean value of cortisol response being higher in the morning than in the afternoon (Figure 8b).

Further measuring the cortisol concentration longitudinally in healthy subjects (*n* = 3) shows reproducible patterns of the sweat cortisol variation in some body locations obtained where it was monitored on random days over a month period (Figure 8c). Knowing that cortisol has fluctuation during weekdays,^[^ ^8g^ ^]^ the sweat cortisol level measured from two opposite body sides between the first day of a weekday (Monday) and the last day of a weekday (Friday) was further analyzed for different body locations (Figure S10, Supporting Information). A relative increase in the cortisol of the armpit sweat from Monday to Friday is seen for all participants and appeared to be profound compared to other body locations.

#### 2.4.3 Cortisol Concentrations and Human Stress Levels

Cortisol plays a significant role in organizing the body's response to physiological and psychological stressors.^[^ ^22b^ ^]^ There are physiological and psychological methods for stress detection,^[^ ^31^ ^]^ but few studies correlated the cortisol concentrations and stress levels (see Table S9, Supporting Information). For instance, the result of a physiological method for the salivary cortisol previously subjected workers' stress levels to different stress levels equivalent to 0.5 to 4 ng mL^−1^ of salivary cortisol.^[^ ^32^ ^]^

**Figure 9:**
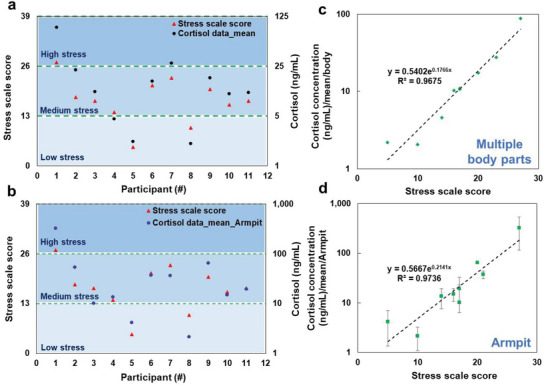
Pairing the stress levels assessed with the perceived stress scale (PSS) test and their corresponding sweat cortisol concentrations in the individual and population levels: a) mean sweat cortisol measurement and their correlated stress score for different participants and b) the relation of sweat cortisol with a stress score of all body locations in the population level. c) Mean sweat cortisol measurement and their correlated stress score for different participants and d the relation of sweat cortisol with a stress score of armpit locations only in the population level. From the PSS test, scores 0–13 refer to low‐stress levels, 14–26 refer to medium stress levels, and 27–40 refer to high‐stress levels.

Herein, to correlate the sweat cortisol concentration to the general psychological stress levels, the perceived stress scale (PSS) test scores (see Section S8, Supporting Information) were compared to their corresponding sweat cortisol concentrations for the cortisol value averaged across multiple body locations (**Figure** **9**a) and the armpits (Figure 9b), separately. The resulting sweat cortisol concentration for multiple body locations ranges from 1 to 5 ng mL^−1^ corresponding to low‐stress levels, 5 to 25 ng mL^−1^ corresponding to the medium stress level, and 25 to 125 ng mL^−1^ for high‐stress levels. These ranges, however, differ for the mean value of only armpits with wider ranges but better compatibility with the PSS test results, with 1–10 ng mL^−1^ for low‐stress levels, 10–100 ng mL^−1^ for medium‐stress levels, and 100–1000 ng mL^−1^ for high‐stress level. The apocrine areas, such as the axillary areas are reported to be responsive to emotional sweating and the apocrine glands are activated by adrenergic stimulation and strongly respond to emotions such as stress.^[^ ^33^ ^]^ Therefore, armpits can be one of the most reliable body locations for future real‐time monitoring of stress levels in different populations because of i) a considerable change in cortisol levels compared to other body locations and ii) better compatibility with the general stress scale tests.

The greater stress an individual experiences over a certain period, the higher the basal cortisol concentration generated in the body.^[^ ^24^ ^]^ The results of sweat cortisol concentration versus stress level (Figure 9c,d) show an exponential relationship between the sweat cortisol values (both the whole body and armpits) and the stress scores. Therefore, cortisol secretion levels are correlated to psychological stress in humans. Such a nonlinear and differential relationship between human stress levels and salivary cortisol concentrations has also been reported in another study.^[^ ^34^ ^]^ In addition, cortisol concentration responds to the induction of physical stress in the body.^[^ ^35^ ^]^ This was detectable for human participant #1, who expressed anxiety issues over a long period, leading to a high basal cortisol concentration. Other subjects examined showed medium ranges of stress levels, although a larger cohort study is needed in future studies to address the discrepancies.

In summary, the wearable MicroSweat patch is a self‐operated and electronic‐free microfluidic system fabricated using a scale‐compatible manufacturing process. It provides a key enabling feature for routine stress monitoring in sports physiology and digital health monitoring. The MicroSweat patch enabled measuring sweat cortisol levels in different body locations, with the most predictive cortisol concentration detectable in the armpits. MicroSweat gives lots of flexibility in the design of human experiments. It also signifies individualizing and personalizing, along with a general view of the cortisol data for longitudinal and daily effects. Sweat cortisol concentration was shown to be higher in apocrine areas versus eccrine regions, more elevated in females (mean value of 55 ng mL^−1^) than males (mean value of 13.7 ng mL^−1^), with larger distribution with the age of below 30 than beyond 30, and with no significant variation between sides of the body. The static increase in the cortisol level was shown to be an indicator of the stress level with severe stress, corresponding to a mean cortisol concentration of 25–125 ng mL^−1^ for the multiple‐body parts and 100–1000 ng mL^−1^ for the armpits. Besides a high degree of correlation between the literature knowledge on cortisol concentration and the MicroSweat data, while considering age and sex differences, it is merited to have further studies on larger cohorts to explore the value of MicroSweat and evaluate the potential benefit of measuring stress levels using the cortisol concentrations of the armpit sweat. Table S9, Supporting Information, shows that compared to the few flexible sensors and/or microfluidic devices currently in the literature^[^ ^8^, ^36^ ^]^ for either direct/indirect stress monitoring in sweat, this work presents continuous sweat flow platform and comprehensively quantified sweat cortisol response regionally, individually including sex, and age, and personally along with daily and longitudinally monitoring considerations. In addition, for the first time, a correlation between sweat cortisol ranges and stress levels is found that is distinguished for regional body locations. These findings can be an essential basis for the future detection of human stress levels using wearable microfluidic and sensor devices. Since there is a strong empirical correlation between serum and sweat cortisol, exciting opportunities are offered for non‐invasive dynamic stress monitoring.^[^ ^8^, ^37^ ^]^

## 3 Experimental Section

### Materials

Cellulose papers (CFP42‐457) were purchased from STERLITECH., Inc, USA as the wicking pads. The flexible TPE layer (9984 Diagnostic Microfluidic Surfactant Free Medical Hydrophilic Film), biocompatible adhesive (Medical Tape 1522, Double Sided Transparent Polyethylene), transparent acrylate adhesives (Microfluidic Diagnostic Tape, 9969 Adhesive Transfer Tape), and a waterproof adhesive layer (Tegaderm Transparent Film Dressing) were provided by 3M, Co, USA. The PSA (AR care @8939 as a polyester film with a double‐sided medical pressure‐sensitive adhesive) was purchased from Adhesives Research., Inc, USA. The lateral flow nitrocellulose (Vivid 90 LFNC, VIV92503R, the wicking rate of 70–110 s cm^−4^) was provided by PALL., Co, NY, USA, and the microfiber glass (Grade 121,) was supplied by Ahlstrom‐Munksjö., Co, Finland. The Human Cortisol ELISA Kits were purchased from two suppliers: LS‐F10024 purchased from Life Span Biosciences., Co, USA, and ab108665 purchased from Abcam., Ltd, Canada.

### Fabrication of MicroSweat

A laser cutter (Trotec Speedy 360 FLEXX 80 W CO2 and 30 W Fiber) was used to pattern the microfluidic network in different fluidic layers and to create the desired design on the fibers. Cellulose papers as the wicking pads were used to absorb the sweat from the skin and conduct it into the inlets of the MicroSweat. The bottom layer of the fluidic layers was a 10 µm flexible TPE layer with inner hydrophilic and outer hydrophobic sides. The hydrophilicity of the TPE stems from the surfactant‐free treatment, which makes the surface intact to various biomarkers. The second and third layers included two layers of 80 µmPSA. The microfluidic network was designed, and laser cut in the PSA layer of MicroSweat, comprising capillary microchannels, valves (stop and delay valves), inlet channels, vents, and storage chambers. The upmost fluidic layer was another TPE layer with inner hydrophilic and outer hydrophobic sides. The storage fibers, including nitrocellulose and microglass, were placed into the storage chambers. The lateral flow nitrocellulose absorbs the low volume of sweat (1–5 µL) while the microfiber glass absorbs a high volume of sweat (20–120 µL), for fibers with a specific size in this study. To create hydrophobic surfaces on the top and bottom surfaces of the storage chambers and cover specific parts of the hydrophilic sides, transparent acrylate adhesives (25 µm thick) were placed on top of the TPE layers. Following the alignment and assembling all the layers manually, the absorbent fibers were embedded into the inlets. Finally, the MicroSweat was gently attached to the skin using the biocompatible adhesive and sealed from the surrounding environment using a waterproof adhesive layer.

### Characterization of the MicroSweat Patch, Sample Collection and Storage

Optical microscopy (Zeiss Inverted Microscope (Axiovert, BF)) was used to analyze the dimensions of the laser‐cut MicroSweat patch. An automotive fluorescent microscopy test was run using Cell Imaging – Microscopy (Eclipse Ti2‐E Nikon, Okolab, Japan) to examine the saturation extent of the storage fibers within the storage chamber. The patch was tested with Rhodamine B fluorescent dye (CAS # 81‐88‐9; Millipore Sigma, Inc, USA) in the water (molecular weight of 479 g mol^−1^ close to cortisol molecular weight, i.e., 362 g mol^−1^). Upon exciting to the dye with ultraviolet (UV) light under the microscope, the emitted fluorescent color was recorded as the light intensity over time. The ELISA tests were detected using a full‐wavelength microplate reader (SpectraMax i3x w/injectors, Co., USA). The contact angles of the hydrophilic and hydrophobic surfaces were measured using an automated system and a contact angle goniometer (Drop Shape Analyzer, DSA100, Kruss) following the dispensing of 6 µL droplets of artificial sweat on the sheets.

### Numerical Modeling of the Sweat Capillary Flow in MicroSweat

To analyze and optimize the sweat capillary flow in the complex microfluidic network of MicroSweat, the electric circuit analogy approach using the dynamic contact angle Bracke model was employed, given the similarity between Hagen–Poiseuille's fluid flow law in microchannels and the Ohm's law Equation.^[^ ^38^ ^]^ To use an electric circuit analogy, the assumption of laminar, viscous, and incompressible fully developed fluid flow was considered for the sweat flow in the microchannels. Furthermore, the microchannels' end effects, surface roughness, and the effect of gravity were ignored.^[^ ^39^ ^]^ Ohm's law describes the relationship between voltage drop (*V*), electrical current (*I*), and resistance (*R*_E_) per equation *V* = *R*_E_ *I*. Similarly, Hagen–Poiseuille's fluid flow law is used for fluid flow in a microchannel to correlate between volumetric flow rate Q, capillary pressure difference at its extremities ∆*p*, and hydraulic resistance of the channel *R*_H_ as ∆*p* = *R*_H_ *Q*. For a rectangular microchannel with the width *w*, height *h*, and length *L* and with fluid viscosity of *µ*, the hydraulic resistance is calculated from Equation (1):^[^ ^38^ ^]^

(1) $$R_{H}=\frac{12\mu L}{wh^{3}\left[1-\frac{192h}{\pi^{5}w}\sum_{n=1,3,5}^{\infty }\frac{1}{n^{5}}\tanh\left(\frac{n\pi w}{2h}\right)\right]}$$

For a rectangular microchannel, the capillary pressure jump across the liquid–gas interface is expressed as Equation (2):^[^ ^35^ ^]^

(2) $$\Delta p=-\gamma \left[\frac{\cos\theta_{t}+\cos\theta_{b}}{h}+\frac{\cos\theta_{l}+\cos\theta_{r}}{w}\right]$$

where *γ* is the surface tension of the liquid in the microchannel, and *θ*_*t*_, *θ*_*b*_, *θ*_*r*_, and *θ*_*l*_ are the top, bottom, right, and left static contact angles of the liquid with four corresponding microchannel walls. The static contact angle during the movement of the liquid over the surface should be modified to a dynamic contact angle^[^ ^40^ ^]^ as Equation 3.

(3) $$\frac{\cos\theta_{s}-\cos\theta_{d}}{\cos\theta_{s}+1}=2Ca^{0.5}$$

where *Ca* is the capillary number defined by *Ca* = *µV*/*γ*, with *V* is the velocity of the interface of the liquid and the air. The Bracke model was compared with experiments and validated in Section S3, Supporting Information. The ImageJ software and the Canon digital camera (EOS 5DSR) were employed to extract the sweat flow data from the experimental tests. One critical parameter of the sweat collection assays is the sequential filling of the side channels. To investigate this criterion, the volume of fluid (VOF) model^[^ ^41^ ^]^ in ANSYS FLUENT 2021R1 is used to model the laminar two‐phase flow inside the main and side channels. This model measures the volume fraction of the water‐liquid or liquid‐air interface position (IP) in hydrophobic side channels (Section S6, Supporting Information).

### Sweat Collection from Human Subjects

Sweat collection was inducted on human subjects during physical exercise and based on their mental stress or exposure to warm and humid environments (e.g., a sauna) or iontophoresis.^[^ ^42^ ^]^ The last three methods either require access to specialized facilities and unique setups or involve pain and/or inflammation due to electric current at the skin interface, limiting their use for non‐intrusive needs of sample collection. In this study, sweating is induced using physical exercise via a 30–60 min treadmill walking at low speed (level 5) while wearing the MicroSweat patches on different body locations, including hands, armpits, foreheads, and backs. It takes about 10 ± 5 s to mount the wearable MicroSwrat patch on the skin. Sweat collection test was conducted on eleven healthy volunteers (five males and six females). The treadmill walking test continued until all storage chambers are filled with sweat, easily identifiable by a color change in the wet fiber. Prior to mounting MicroSweat, the skin was cleaned with 70% ethanol pads, rinsed, and dried thoroughly.

As reported by one study,^[^ ^8e^ ^]^ cortisol reaches its maximum right after exercising and decreases thereafter 2 h. On the other hand, another study^[^ ^8g^ ^]^ concluded that the sweat cortisol increases progressively and reaches the highest level after 40 min, decreasing after that, until it recovers at 50 min of continuous biking. To analyze the cortisol biomarker in sweat samples in this study, the samples collected from a mixture of multiple storage fibers of MicroSweat filled within 40 min exercising were retrieved and with equal volume tested to be consistent for all subjects. Following the sweat collection, MicroSweat was easily peeled off from the skin. The stored fibers were retrieved from the patches, placed in a storage vial, and immediately transferred to the freezer at ‐80 °C until processing. The sweat liquid was recovered from the wet fibers by centrifuging the storage fibers at 11 000 rpm for 5 min.

The stress level of participants was assessed using two different approaches: 1) the Perceived Stress Scale (PSS) test^[^ ^43^ ^]^ collected before the exercise study and one more time one month after the exercise, and 2) sweat cortisol measurement (see Section 2.4.) from the sweat samples. The level of stress was estimated using the PSS score and cortisol concentration. The stress scores range from 0 to 40, with scores < 13 corresponding to low‐stress levels, between 13 and 26 corresponding to the moderate stress level, and above 26 corresponding to the severe stress level.

### Cortisol Concentration Measurements using the Gold Standard Method

The sweat samples collected from the volunteers were subject to cortisol concentration measurement using an enzyme‐linked immunosorbent assay (ELISA). The responses of the ELISA reaction were first tested with spiked cortisol solutions in the water and artificial sweat. A 0.1 mg mL^−1^ cortisol solution in the water was serially diluted with the water down to 1.0 µg mL^−1^ concentration. This solution was further diluted in both water and artificial sweat to make cortisol solutions in both solvents to compare against the cortisol calibrator solutions provided in the cortisol ELISA kit. Figure S11, Supporting Information, shows no deviation between the ELISA standard curve and the calibration curve for the spiked artificial sweat sample with pH = 6, confirming the minimal effect of physiologically relevant sweat pH in measuring sweat cortisol concentration. The analytical procedures were followed based on the recommended protocols provided by the suppliers of the cortisol ELISA kits. A urinary cortisol ELISA kit was selected due to its wide cortisol detection range of 0.47–200 ng mL^−1^, meeting the needs of human skin cortisol concentration measurement, and previously used in other reports.^[^ ^23^ ^]^ For those samples that had cortisol with concentrations above 200 ng/mL, a salivary cortisol kit with a dynamic detection range of 10–500 ng mL^−1^ was employed to quantify the cortisol level. According to the ELISA kit guide, the sweat samples were centrifuged for 10 min at 4000 rpm to remove particulates and the supernatant before assaying.

### Statistical Analysis

Data analysis was conducted in R v.4.1.1. The significance level for all statistical tests was set at 0.05. A two‐tailed *t*‐test was used to determine mean differences of total and regional sweat between different groups of sex, age, and side of the body in measures of cortisol data. *F*‐test was used to compare the standard deviations of two sample groups. The detailed values of t‐test and F‐test were reported in Tables S3–S8, Supporting Information. The cortisol data analysis was based on the availability of the dataset for different individuals, and all the figures were plotted via R. The error bars are shown in Figure 3c,d, and Supporting Figures. S1, S3, and S6, Supporting Information are related to the experimental testing of 10 different MicroSweat patches. The error bars in human trials of Figures 5b and 6 are connected to three tests.

## Conflict of Interest

S.S, R.S, D.R, J. S.B, and A.S.N filed a US provisional patent No. 63/229,216, based on this work. The other authors declare no competing interests.

## Author Contributions

S.S. conceived the idea and design, led the research, conducted experiments, characterizations, fabrication, and data acquiring and analysis, wrote the original manuscript; R.S. and M.H. contributed to the design, and fabrication of the device; A.Z. performed the numerical modeling, S.M. helped with the statistical analysis, E.M. and R.S. helped with chip fabrications, sample collections and testing, D.R. helped with physiological insights, and experimental resources; J.S.B and A.S.N helped with the idea and design of the device, supervised the experimental work and data analysis. All authors reviewed the manuscript.

## Supporting information

Supporting Information

Supporting Information

Supplemental Movie 1

Supplemental Movie 2

## Data Availability

The data that support the findings of this study are available from the corresponding author upon reasonable request.
