# Supplementary material for: MicroSweat: A Wearable Microfluidic Patch for Noninvasive and Reliable Sweat Collection Enables Human Stress Monitoring
Source: Adv Sci (Weinh). 2022 Dec 3;10(7):2204171. doi: 10.1002/advs.202204171 (PMC9982588; doi:10.1002/advs.202204171)
Supplement: Supplementary file 1 — Supporting Information [file ADVS-10-2204171-s002.pdf]

ADVANCED SCIENCE
Open Access

Supporting Information

for *Adv. Sci.*, DOI 10.1002/advs.202204171

MicroSweat: A Wearable Microfluidic Patch for Noninvasive and Reliable Sweat Collection Enables Human Stress Monitoring

Shaghayegh Shajari, Razieh Salahandish, Azam Zare, Mohsen Hassani, Shirin Moossavi, Emily Munro, Ruba Rashid, David Rosenegger, Jaideep S. Bains and Amir Sanati Nezhad**

Supporting Information

MicroSweat: a wearable microfluidic patch for noninvasive and reliable sweat collection enables human stress monitoring

Shaghayegh Shajari[a,b,c], Razieh Salahandish[a,c], Azam Zare[a], Mohsen Hassani[a], Shirin Moossavi[a,d,e], Emmi Munro[f], Ruba Rashid[g], David Rosenegger[h], Jaideep S. Bains[b*], and Amir Sanati Nezhad[a,c*]

[a] BioMEMS and Bioinspired Microfluidic Laboratory, Department of Biomedical Engineering, University of Calgary, Calgary, Alberta T2N 1N4, Canada

[b] Stressynomics, Hotchkiss Brain Institute, Cumming School of Medicine, University of Calgary, Calgary, Alberta T2N 1N4, Canada

[c] Department of Mechanical and Manufacturing Engineering, University of Calgary, Calgary, Alberta T2N 1N4, Canada

[d] Department of Physiology and Pharmacology, University of Calgary, Calgary, Alberta T2N 1N4, Canada

[e] International Microbiome Centre, Cumming School of Medicine, Health Sciences Centre, University of Calgary, Calgary, Alberta T2N 1N4, Canada

[f] Department of Chemical and Petroleum Engineering, University of Calgary, Calgary, Alberta T2N1 N4, Canada

[g] Department of Civil Engineering, University of Calgary, Calgary, Alberta T2N1 N4, Canada

[h] SenseSi Co., Calgary, Alberta, Canada

This PDF file includes:

Tables S1 to S9

Figures S1 to S11

Table S1. Optimum laser cutter parameters for laser cutting different layers of MicroSweat

Materials	Power (Hz)	Speed (rpm)	Number of runs
Pressure sensitive adhesive (PSA)	60	50	2
Polyethylene terephthalate (PET)	42	35	2
Fibers	60	35	5
Hydrophobic adhesive	55	34	1
Skin adhesive	58	35	5
Waterproof adhesive	52	35	3

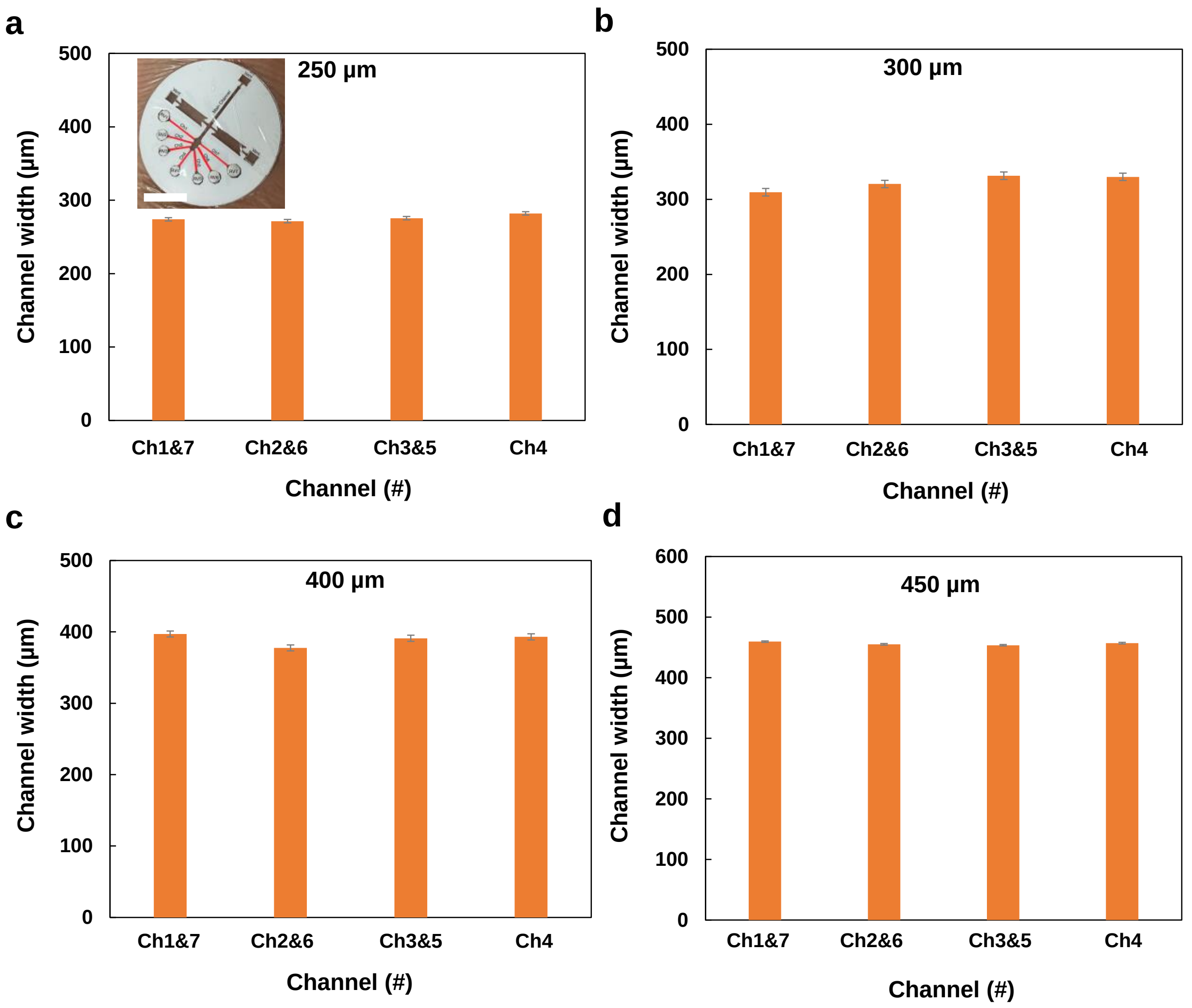

Figure S1. Demonstration of repeatability of laser cutting process. The repeatability of the optimized laser cutting parameters for fabrication of MicroSweat with different channel widths of a 250 µm (Scale bar: 10 mm), b 300 µm, c 400 µm, and d 450 µm.

SI.1. Geometry information of the MicroSweat patch

Figure. S2 shows additional details of specific embodiments of the MicroSweat patch, including a diameter for 30 mm and the channel widths and depths for 100–200 µm and 80 µm, respectively. Different channel widths ranging from 100-200 µm were used for Ch1-Ch7, and the width of 700 µm was used for Ch8. The inlet reservoirs RV3, RV4, and RV5 have a diameter of 2 mm, RV2 and RV6 have a diameter of 2.5 mm, and RV1 and RV7 have a diameter of 3 mm.

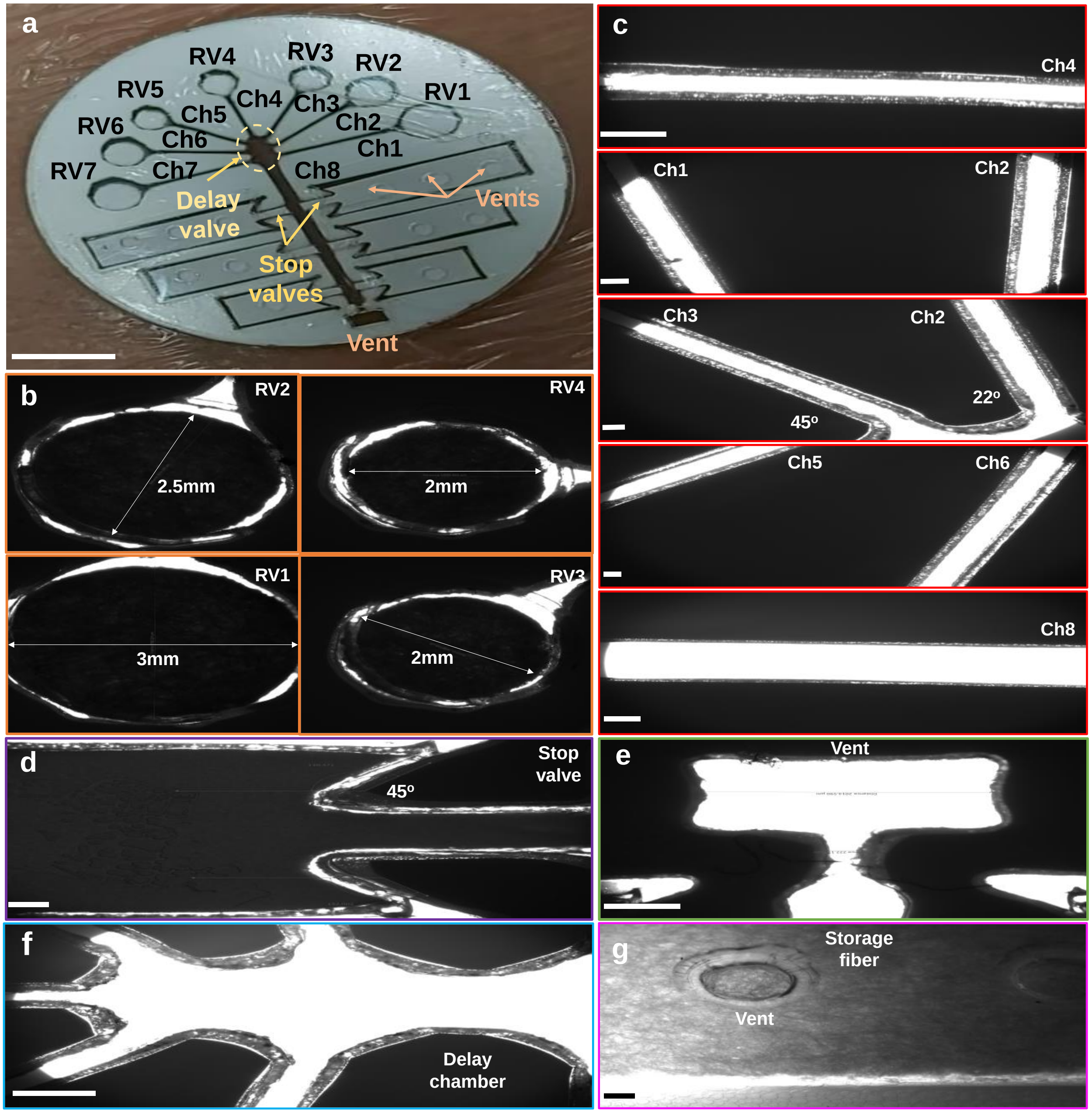

Figure S2. Construct of MicroSweat and its optical imaging. a Indication of different components of the epidermal MicroSweat patch. Optical images of the b inlet reservoirs (RV), c channels (Ch), d stop valves, e the main vents, f delay chamber, and g storage fibers and micro-reservoir vents. Scale bars: a 5 mm, c civ d e f 500 µm, ci cii ciii g 100 µm.

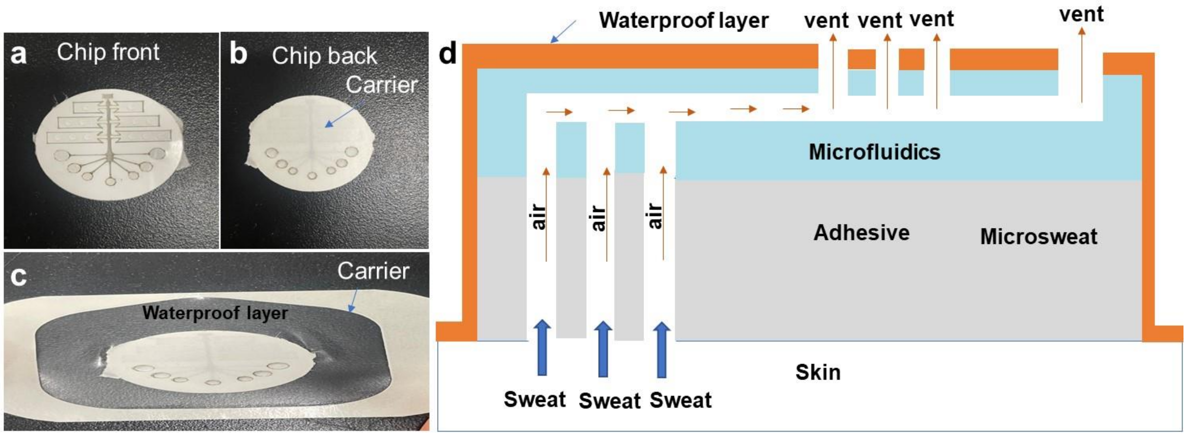

Figure S3. The images of MicroSweat as a single integrated unit containing several assembled layers. a Front view, b back view without a waterproof layer, and c back view showing the waterproof layer. d Schematic side view of MicroSweat showing the sealing condition and the air release through the channel network.

SI.2. Sealing condition of MicroSweat filled with the sweat under different bending and stretching tests.

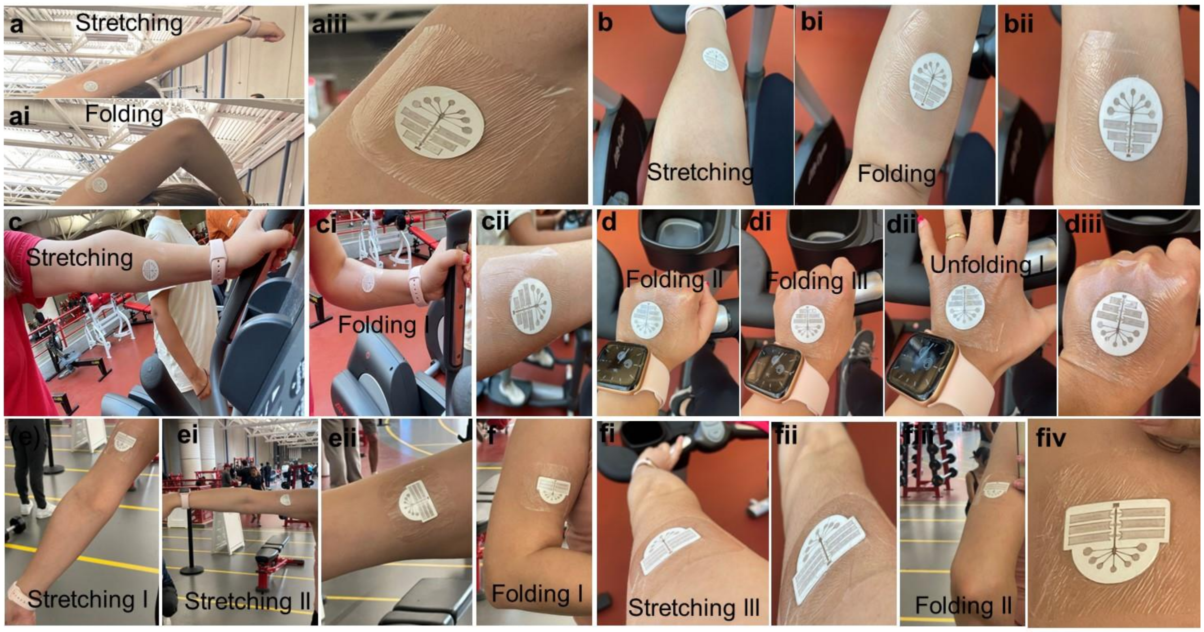

Figure S4. MicroSweat filled with the sweat subject to different stretching, releasing, folding and unfolding modes for, a-ai The top position of the upper arm and aii magnified view. b-c Forearm position and bii and cii their magnified view. d-dii Hand position and diii magnified view. e-ei The bottom position of the upperarm and eii magnified view. f-fiii Back of the forearm position and fiv magnified view, all confirming no leakage in MicroSweat.

Table S2. Geometric information of laser-cut microchannels of MicroSweat

Resistance	Width (μm)	Length (mm)	Height (μm)	Static contact angle (Artificial Sweat)	
				Top & Bottom	Left & Right
1,3	200	6	80	31.5	67.4
2	200	5.5	80	31.5	67.4
4	730	0.6	80	31.5	67.4
5,6	200	8	80	31.5	67.4
7	1000	0.46	80	31.5	67.4
8,9	200	9.4	80	31.5	67.4
10	500	4	130	31.5	67.4
11,12	200	9.4	130	120	67.4
13	500	11	130	31.5	67.4

SI.3. Evaluation of the Bracke model in the numerical modeling

To evaluate the Bracke model in this numerical modeling, the water flow in a straight microchannel with the width and height of 300 and 80 µm, respectively, was simulated and compared to the experimental data. The water-air interface position and the liquid velocity versus time are shown in **Figure. S5a, b**. The numerical results agree with the experimental data of capillary flow in straight channels where the main deviation is observed at the unsteady flow condition at the beginning of the channel caused by the uncertainties in accurately measuring the channel dimensions and the dynamic contact angles. Despite the unavoidable overestimation of the liquid interface velocity at the channel's beginning, the numerical model is well predicting the capillary liquid flow in the microchannel.

When the liquid is dispensed into the inlet, capillary force draws the liquid inside the channel, accompanied by increased flow resistance along the channel length. This reduces the liquid flow rate, but the flow never stops as long as the air-liquid interface exists within the channel (**Figures S5a**). Figure S5b shows this phenomenon for a long channel (20 mm in length) where the flow rate decreases from 200 mm/s to 30 mm/s. However, the channel length in MicroSweat is much

smaller (3-6 mm in length) than that simple capillary channel, leading to a considerably lower decrease in the flow rate for only 30% of the initial flow rate, i.e., down to 120 mm/s. Although adding the sweat gland force to the capillary force further contributes to increasing the sweat flow rate, the capillary pressure alone is sufficient to fill up the MicroSweat patch.

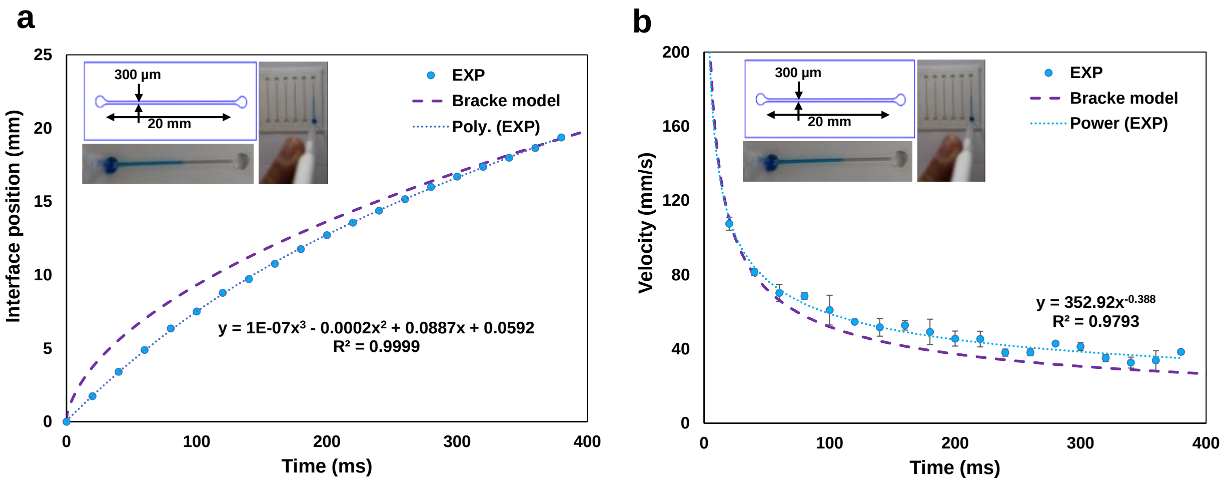

Figure S5. Verification of the Bracke numerical modeling. A comparison between experimental results and numerical modeling for a the interface position of liquid over time, and b liquid velocity over time for one simple straight channel. The inset images in the a and b show the experiments testing 10 different channels using water colored by blue dye.

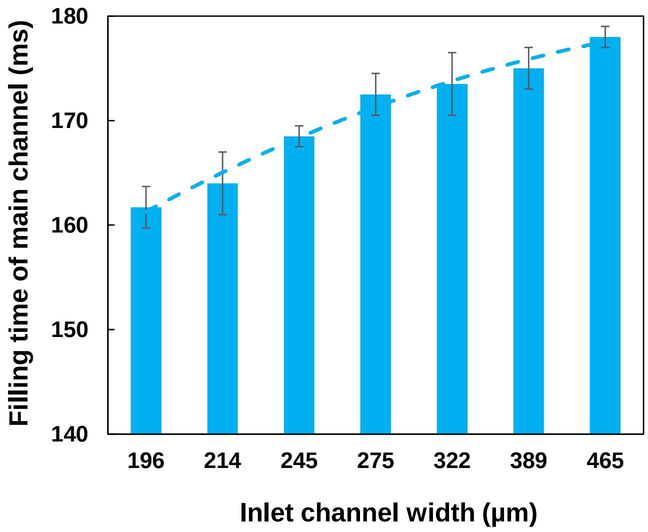

Figure S6. The effect of inlet channel width on the filling time of the main channel at the outlet position in the experimental tests.

SI.4. Sweat evaporation rate in Microsweat during human testing

The mass of water evaporated during human testing of MicroSweat was calculated for a minimum of 10 min exposure time for filling the last fiber and a maximum of 40 min exposure time for filling the first fiber. According to Langmuir's free evaporation equation in the vacuum, the mass loss rate at a given temperature T is obtained using the equation below [50, 51].

$$-\frac{dm}{dt} = \alpha S P_v \sqrt{\frac{M}{2\pi RT}}$$

where dm/dt is the rate of mass loss (kg.s−1), S is evaporating surface area (πd2/4), d is the diameter of the orifice, which is the vent diameter in this case, Pv is vapor pressure (Pa), M is the molecular weight of the vapor of the evaporating compound (kg mol−1), R is the gas constant (JK^{-1} mol^{-1}), T is the absolute temperature, and α is vaporization coefficient. In a vacuum, α is assumed to be 1, but as commonly found in TG experiments, α has a significantly different value in a flowing gas atmosphere. We consider α as 5.8-6.6 × 10^{-5} (Pa^{-1} s^{-1} m^2) according to the studies with significant deviation from vacuum conditions. Therefore, the mass loss rate was estimated to be about 4.4 × 10^{-10} g. s^{-1}, resulting in a mass loss value of 0.001 mg (0.001 μL) during a maximum of 40 min human tests. This amount can be compared to the amount of sweat absorbed by the

storage fibers for a maximum volume of 20 µL (~ 20 mg) for microglass fibers and 1µL (~ 1 mg) for nitrocellulose fibers, showing that there is almost no sweat loss during the experiment. The assumptions here are: i) 99% of the sweat is water,[1] and ii) the rate of mass loss is calculated for the bulk of water.[2]

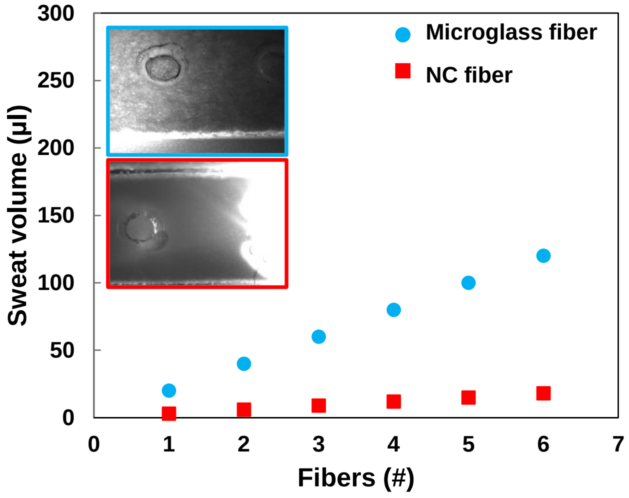

Figure S7. Comparing the capacity of nitrocellulose fibers and glass microfibers' ability to absorb the sweat quantified using optical imaging.

SI.5. Saturation test of the fibers

A fluorescent dye dissolved in the water was injected into MicroSweat (using multiple pipettes delivering simultaneously to all inlets) until the fibers absorbed the dyed water (**Figure. S8a, b**). The transparency of this chip guarantees that all fluorescent light detected by the fluorescent microscope is stemmed from either the dyed water or the fiber without being affected by light reflection from PSA. The fibers also showed that they have no or minimal autofluorescent signal. **Figure. S8c, d** shows the images of the fibers in the vertical or horizontal positions when saturated with the fluorescent dye. The plots of fluorescent intensity versus time for six different fibers are shown in **Figure. S8e, f**. These graphs indicate the saturation points for all the fibers realized by the plateau behavior of the color intensity in both vertical and horizontal directions.

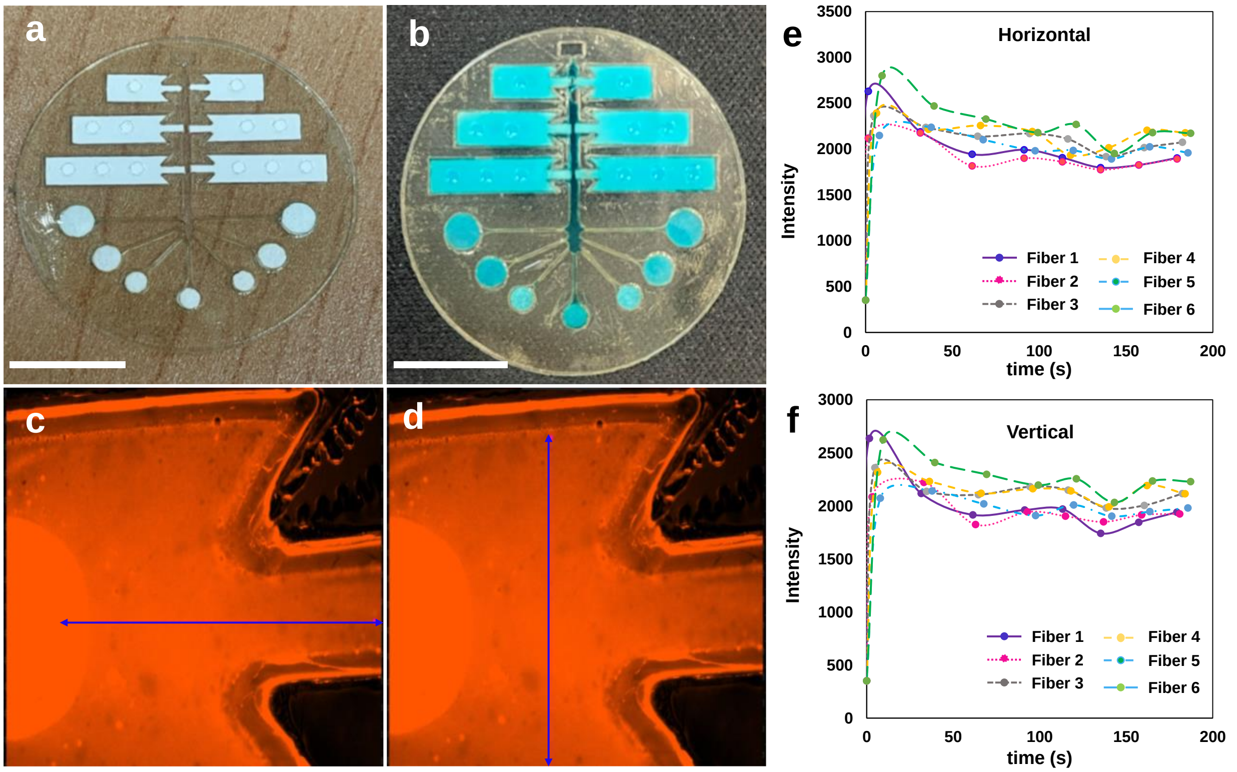

Figure S8. Saturation analysis of the storage fibers in the MicroSweat chip. a Characterizing the saturation level of storage fibers using transparent MicroSweat. b Injection of a fluorescent dye into the device and measuring the fluorescent intensity under the microscope. c The color intensity image in the horizontal direction and d vertical direction. e The corresponding fluorescent intensity of the storage fiber at the saturation points along its length in the horizontal direction and f the corresponding fluorescent intensity of the storage fiber at the saturation points along its width in the vertical direction. Scale bars: a b 10 mm.

SI. 6. Order filling of MicroSweat simulated using a computational model

We further conducted a computational simulation to support the sequential filling of the storage chambers. **Figure S9** shows the results of the ANSYS simulation, with two assumptions considered: a) the storage chambers are removed in this simulation, and b) only one channel collects the sweat at the inlet. Figure S9a shows the visual two-phase volume fraction indicating that the delay valves are activated sequentially from the bottom to the top. Figure S9b shows the sequence of filling the interfacing channels from the bottom to top and from left to right, indicated by the change in position of the air-liquid interface versus time. The order of filling the chambers is from 1L (350 μm in width for the valve) to 1R (250 μm in width), followed by 2L (250 μm in width) to 2R (175 μm in width), and finally 3L (175 μm in width) to 3R (125 μm in width). A

similar sequence of filling was also confirmed during human testing with the MicroSweat patch. Given the assumptions implied, only the sequence but not the filling time values are comparable between the experimental data and numerical simulations in this simulation.

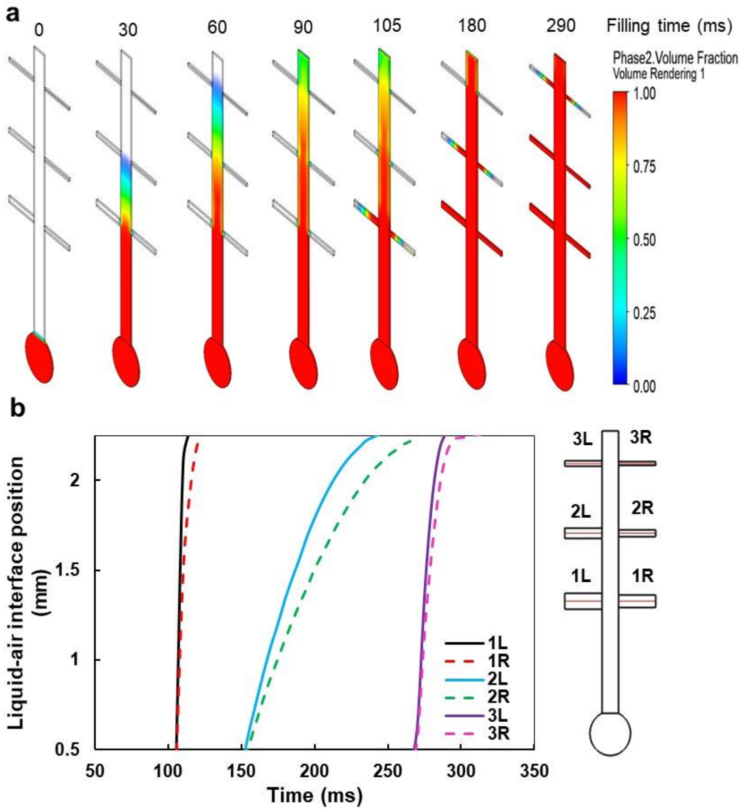

Figure S9. Numerical simulation of the water-air flow along the channel network of the MicroSweat design. a) The two-phase volume fraction and b) the position of the water-air interface at different time points.

SI.7. T-test and F-test

F-test and T-test are the two statistical tests used for hypothesis testing. They assist in deciding whether to accept or reject the null hypothesis. The F-test is usually used to compare the two standard deviations of two samples and check the variability. F-test is carried out as a single-sided test as variance cannot be negative. Under the null hypothesis, the F-statistic follows the Snedecor's F-distribution. The F-test can be applied on a large, sampled population. T-test is used to compare the means of two different sets. Under the null hypothesis, T-test is based on Student

t-distribution. T-test is used when the sample population is small and the sample on which the test was applied remains the same. We used F-test and t-test to investigate the effect of sex and age differences and body sides on cortisol concentrations measured in different body locations.

Table S3. The effect of sex differences on the sweat cortisol concentration, examined using F-test and t-test.

Sex	count	mean	SD		
**Female**	68	55.07	137.79		
**Male**	54	13.73	50.79		
**F-test**	**F**	**Num df**	**Denom df**	**p-value**	**Ratio of variance**
	7.36	67	53	5.21e-12	7.36
**T-test**	**t**	**df**	**p-value**		
	2.09	120	0.038		

Table S4. The effect of sex differences on the sweat cortisol concentration on different body locations, examined using t-test.

**Armpit**			
**T-test**	**t**	**df**	**p-value**
	2.6	41	0.012
**Hand**			
**T-test**	**t**	**df**	**p-value**
	0.87	15	0.35
**Back**			
**T-test**	**t**	**df**	**p-value**
	1.36	47	0.17
**Forehead**			
**T-test**	**t**	**df**	**p-value**
	-0.98	8	0.35

Table S5. The effect of age differences on the sweat cortisol concentrations, examined using F-test and t-test.

Ages	count	mean	SD		
**<30**	75	54.02	137.39		
**>=30**	44	9.52	11.29		
**F-test**	**F**	**Num df**	**Denom df**	**p-value**	**Ratio of variance**
	148.01	74	43	< 2.2e-16	148.00
**T-test**	**t**	**df**	**p-value**		
	2.14	117	0.034		

Table S6. The effect of age differences on the sweat cortisol concentration on different body locations, examined using t-test.

**Armpit**			
**T-test**	**t**	**df**	**p-value**
	3.1	41	0.003
**Hand**			
**T-test**	**t**	**df**	**p-value**
	-2.36	15	0.032
**Back**			
**T-test**	**t**	**df**	**p-value**
	-1.48	47	0.144
**Forehead**			
**T-test**	**t**	**df**	**p-value**
	0.20	8	0.84

Table S7. The effect of body sides on the sweat cortisol concentration, examined using F-test and t-test.

**body Sides**	**count**	**mean**	**SD**		
**Left**	26	72.47	157.16		
**Right**	35	66.63	144.80		
**Upper**	23	4.44	6.75		
**Lower**	25	5.04	5.45		
**F-test Left/right**	**F**	**Num df**	**Denom df**	**p-value**	**Ratio of variance**
	1.18	25	34	0.64	1.18
**F-test Upper/lower**	1.54	22	24	0.31	1.54
**T-test Left/right**	**t**	**df**	**p-value**		
	0.15	59	0.88		
**T-test Upper/lower**	-0.34	46	0.73		

Table S8. The effect of different body sides on the sweat cortisol concentration for different body locations, examined using t-test.

**Armpit**			
**T-test**	**t**	**df**	**p-value**
	-0.081	41	0.93
**Hand**			
**T-test**	**t**	**df**	**p-value**
	-0.87	15	0.39

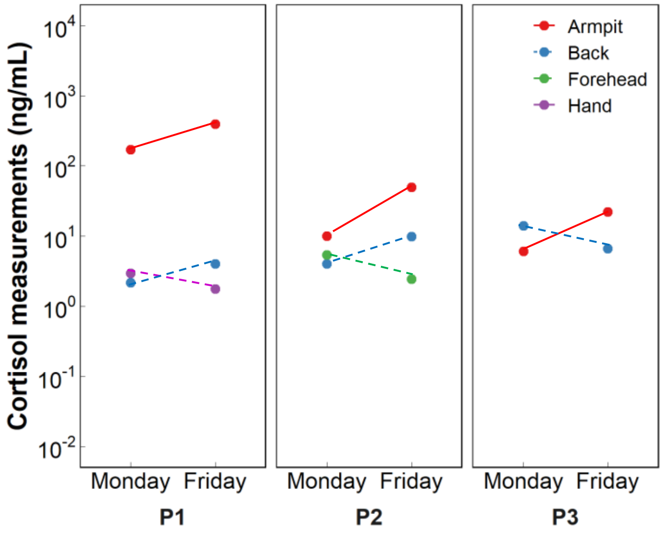

Figure S10. Personalized weekly variation of sweat cortisol for three healthy participants (P1-P3) and different body locations. Sweat cortisol variation a on Monday and b on Friday.

Table S9. Stress monitoring in real sweat using flexible sensors/microfluidic devices

Flexible sensor/ microfluidic	Sex/ age considerations	Chrono sampling	µfluidic Material	Cortisol ranges in real sweat	Correlation with stress (long term/short term)	Regional consideration	Stress level	Personalized information	Compatibility with scale-up fabrication	Ref
**Flexible sensor/ patch**	No	No	PDMS[a)]	NP[e)]	No	NP	NP	NA	NP	60
**Flexible sensor**	No	No	NA[b)]	86-200 nM	Yes-short term-cold test	Fingertip	NP	5 individuals/daily monitoring	NP	22
**Flexible sensor**	No	No	NA	NP	No	NP	NP	1 Individual	NP	61
**Flexible sensor/ microfluidic**	No	No	PDMS	NP	No	Forehead	NP	3 Individual/daily monitoring	NP	20
**Flexible sensor**	No	No	NA	8-141 ng/mL	No	Hand	NP	1 Individual/daily monitoring	NP	23
**Flexible sensor**	No	No	NA	NP	Yes-short term-cold test	Forehead	NP	4 Individual/daily monitoring	NP	24
**Flexible sensor/ microfluidic**	No	No	PET[c)]	NP	Yes-short term-social stress test	NP	NP	17 Individual/daily monitoring	NP	62
**Flexible microfluidic**	Yes	Yes	Surfactant-free PE[d)]	(Averaged body parts) 1-125 ng/mL (Armpits) 1-1000 ng/mL	Yes-long term	Forehead, hand, back, armpits, chest and forearm	(Averaged body parts) Low level: 1-5 ng/mL Medium level: 5-10 ng/mL High level: 10-125 ng/mL (Armpits) Low level: 1-10 ng/mL Medium level: 10-100 ng/mL High level: 100-1000 ng/mL	11 Individual/daily/longitudinal/regional	Yes	This work

[a)] PDMS: polydimethylsiloxane, [b)] NA: not applicable, [c)] PET: polyethylene terephthalate, [d)] PE: polyester, [e)] NP: not provided

SI. 8. Perceived stress test

A more precise evaluation of personal stress can be determined by using a variety of instruments developed to measure the stress levels of individuals. A known method of measuring stress levels is the Perceived Stress Scale (PSS) as a classic stress assessment instrument. The PSS, initially developed in 1983, remains a popular choice for understanding different situations affecting human feelings and perceived stress. The PSS questions ask about the feelings and thoughts of the individual during the last month. The subject is asked to indicate how often he/she felt or thought a certain way. Individual scores on the PSS can range from 0 to 40, with higher scores indicating higher perceived stress. Scores ranging from 0-13 are considered low stress. Scores ranging from 14-26 are regarded as moderate stress. Scores ranging from 27-40 are considered high perceived stress.

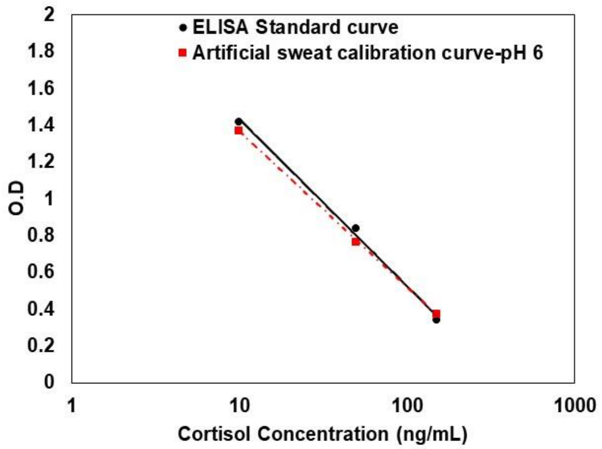

Figure S11. The standard curve of cortisol ELISA assay and the calibration curve used for detection of different concentrations of cortisol in spiked artificial sweat samples.

References

1. Robinson, S.; Robinson, A. H., Chemical composition of sweat. *Physiological Reviews* **1954,** *34* (2).
2. Peng, Y.; Li, W.; Liu, B.; Jin, W.; Schaadt, J.; Tang, J.; Zhou, G.; Wang, G.; Zhou, J.; Zhang, C.; Zhu, Y.; Huang, W.; Wu, T.; Goodson, K. E.; Dames, C.; Prasher, R.; Fan, S.; Cui, Y., Integrated cooling (i-Cool) textile of heat conduction and sweat transportation for personal perspiration management. *Nat Commun* **2021,** *12* (1), 6122.
