# Supplementary material for: MicroSweat: A Wearable Microfluidic Patch for Noninvasive and Reliable Sweat Collection Enables Human Stress Monitoring
Source: Adv Sci (Weinh). 2022 Dec 3;10(7):2204171. doi: 10.1002/advs.202204171 (PMC9982588; doi:10.1002/advs.202204171)
Supplement: Supplementary file 2 — Supporting Information [file ADVS-10-2204171-s003.pdf]

ADVANCED SCIENCE
Open Access

Supporting Information

for *Adv. Sci.*, DOI 10.1002/advs.202204171

MicroSweat: A Wearable Microfluidic Patch for Noninvasive and Reliable Sweat Collection Enables Human Stress Monitoring

Shaghayegh Shajari, Razieh Salahandish, Azam Zare, Mohsen Hassani, Shirin Moossavi, Emily Munro, Ruba Rashid, David Rosenegger, Jaideep S. Bains and Amir Sanati Nezhad**

Supplementary Movie

A Chrono sampling of sweat using MicroSweat

Supplementary Movie 1 shows that sweat runs through the storage fibers in a timely manner, and when the storage fibers are soaked with sweat, they look darker. Therefore, by adjusting the capacity of the storage fibers and the geometry of the microchannels and delay valves, the filling time of the storage fibers and thus their sequence can be controlled. We change the speed of the movie to make it faster for a quick look at the Chrono sampling phenomenon.
